# ADFound: A Foundation Model for Diagnosis and Prognosis of Alzheimer’s Disease

**DOI:** 10.1109/JBHI.2025.3576436

**Published:** 2025-11-01

**Authors:** Guangqian Yang, Kangrui Du, Zhihan Yang, Ye Du, Eva Yi Wah Cheung, Yongping Zheng, Mo Yang, Zoe Kourtzi, Carola-Bibiane Schönlieb, Shujun Wang

**Affiliations:** Department of Biomedical Engineering, The Hong Kong Polytechnic University, Hong Kong SAR, China; School of Computational Science and Engineering, Georgia Institute of Technology, USA; Department of Computer Science, National University of Singapore, Singapore; Department of Biomedical Engineering, The Hong Kong Polytechnic University, Hong Kong SAR, China; Department of Diagnostic Radiology, School of Clinical Medicine, LKS Faculty of Medicine, University of Hong Kong, Hong Kong SAR, China; Department of Biomedical Engineering and Research Institute for Smart Ageing, The Hong Kong Polytechnic University, Hong Kong SAR, China; Department of Biomedical Engineering, The Hong Kong Polytechnic University, Hong Kong SAR, China; Department of Psychology, University of Cambridge, Cambridgeshire, UK; Department of Applied Mathematics and Theoretical Physics, University of Cambridge , Cambridgeshire, UK; Department of Biomedical Engineering, The Hong Kong Polytechnic University; Research Institute for Smart Ageing, The Hong Kong Polytechnic University; Research Institute for Artificial Intelligence of Things, The Hong Kong Polytechnic University

**Keywords:** Alzheimer’s Disease, Dementia, Contrastive Learning, Foundation Model, Multi-modal Learning

## Abstract

Alzheimer’s disease (AD) is an incurable neurodegenerative disorder characterized by progressive cognitive and functional decline. Consequently, early diagnosis and accurate prediction of disease progression are of paramount importance and inherently complex, necessitating the integration of multi-modal data. However, most existing methods are task-specific models that lack generalization ability, addressing only one task at a time and failing to simultaneously assess disease diagnosis and progression. In this paper, we introduce ADFound, the first foundation model for AD that serves as a basis for various downstream tasks, such as diagnosis and prognosis, with high generalization capability. ADFound leverages a substantial amount of unlabeled 3D multi-modal neuroimaging, including paired and unpaired data, to achieve its objectives. Specifically, ADFound is developed upon the Multi-modal Vim encoder by Vision Mamba block to capture long-range dependencies inherent in 3D multi-modal medical images. To efficiently pre-train ADFound on unlabeled paired and unpaired multi-modal neuroimaging data, we proposed a novel self-supervised learning framework that integrates multi-modal masked autoencoder (MAE) and contrastive learning. The multi-modal MAE aims to learn local relations among modalities by reconstructing images with unmasked image patches. Additionally, we introduce a Dual Contrastive Learning for Multi-modal Data to enhance the discriminative capabilities of multi-modal representations from intra-modal and inter-modal perspectives. Our experiments demonstrate that ADFound outperforms state-of-the-art methods across a wide range of downstream tasks relevant to the diagnosis and prognosis of AD. Furthermore, the results indicate that our foundation model can be extended to more modalities, such as non-image data, showing its versatility. The code is available at https://github.com/guangqianyang/ADFound.git.

## I Introduction

**A**LZHEIMER’S disease (AD) is widely recognized as a progressive and irreversible neurodegenerative disorder predominantly affecting the elderly, accounting for over 60% of dementia cases [1], [2]. This condition results in incurable memory loss, cognitive impairment, and functional deficits, ultimately leading to a loss of functional independence [3]. As the global population ages, the prevalence of AD is expected to increase significantly, imposing a substantial burden on healthcare systems and society at large [4]. Therefore, automatic early diagnosis and accurate prognosis of AD are crucial for timely prevention and intervention to mitigate the progressive neurodegeneration and the associated societal burden.

The rapid advancement of deep learning techniques has significantly enabled the creation of high-performance models for various AD tasks [5]–[11]. However, these methods face two key challenges. First, training these models [5]–[10] is often challenging due to the scarcity of annotations in the medical domain, and their generalization ability is limited by the specific task for which they are trained. Second, most models [5]–[9], [11] in the AD domain rely on data from a single modality, which is a stark contrast to the multifaceted approach medical experts use to diagnose and reason about conditions and diseases. In fact, experts typically make decisions using multi-modal data, such as Magnetic resonance imaging (MRI), positron emission tomography (PET) scans, and non-image data, to obtain a comprehensive and reliable understanding of a patient’s condition [12]. Therefore, it is critical to develop a generalized foundation model based on multi-modal data for AD that serves as a versatile and robust base for a wide range of downstream tasks.

Recently, foundation models have demonstrated exceptional generalization capabilities, driven by the availability of extensive medical data [13]–[24]. Representative methods such as RETFound [13], USFM [20], and MIS-SFM [21] successfully pre-train modality-specific foundation models on large-scale unlabelled datasets (e.g., retinal images, ultrasound, CT), achieving remarkable performance across diverse downstream tasks. Additionally, multi-modal foundation models like CONCH [15] and KAD [22] leverage complementary information from image-caption pairs for improved disease prediction in computational pathology and chest radiology. Despite these advances, applying foundation models to AD domain presents three key challenges. First, most foundation models [13]–[20], [22] rely on Vision Transformers (ViTs) as their backbone, which suffer from high quadratic complexity due to self-attention mechanisms, making them inefficient for high-resolution 3D medical images with numerous 3D patches [25]. Second, these models primarily employ pretraining strategies like masked autoencoders (MAE) or contrastive learning [13], [15]–[17], [19], [20], [22]. MAE excels at local detail reconstruction but struggles to capture global discriminative representations, whereas contrastive learning focuses on global relations across modalities. Integrating these approaches could enhance multi-modal representation learning for AD analysis. Third, most multi-modal foundation models [15], [16], [18], [19], [22]–[24] rely on paired multi-modal data, limiting their applicability in real-world scenarios where multi-modal data are often unpaired. Developing models capable of learning meaningful representations from unpaired data is critical to leveraging diverse information and improving robustness and flexibility in AD diagnosis and prognosis.

To address these challenges, we propose ADFound, a pioneering 3D foundation model for multi-modal AD diagnosis and prognosis tasks. ADFound features a multi-modal Vim encoder that leverages Vision Mamba (Vim) blocks to efficiently capture long-range dependencies in 3D neuroimaging data, such as MRI and PET scans, while achieving linear scalability with sequence length to reduce computational demands. AD-Found is developed in two stages: self-supervised pre-training and downstream task adaptation. During pre-training, AD-Found employs an innovative self-supervised learning (SSL) framework that combines a multi-modal MAE for reconstructing 3D masked multi-modal images to capture local relationships and a dual contrastive learning module for robust representation learning. The dual module includes intra-modal contrastive learning to enhance feature discrimination across paired and unpaired modalities and inter-modal contrastive learning to address misalignment within paired modalities. This enables ADFound to generate transferable multi-modal representations for downstream tasks. In the downstream task adaptation stage, the pre-trained ADFound model is fine-tuned for various AD diagnosis and prognosis tasks, including CN vs. MCI vs. AD, CN vs. MCI, CN vs. AD, MCI vs. AD, and sMCI vs. pMCI, ADFound demonstrates superior accuracy and generalization compared to state-of-the-art methods. Furthermore, ADFound can seamlessly incorporate additional data modalities, such as demographic, genetic, and psychological data, to enhance its versatility and predictive performance.

To summarize, our contributions are as follows:

1) We introduce ADFound, the first foundation model pretrained on a large-scale, unlabeled multi-modal neuroimaging dataset with paired and unpaired data, adaptable to a wide range of AD-related tasks.
2) We develop a multi-modal Vim encoder based on Vim blocks to effectively model long-range dependencies in MRI-PET pairs.
3) We propose a novel SSL strategy that integrates dual contrastive learning into a multi-modal MAE for robust representation learning.
4) Extensive experiments validate the superior performance and generalization ability of ADFound across various downstream tasks in AD diagnosis and prognosis.

The rest of this paper is arranged as follows. We delve into related works in Section II. We then elaborate on the technical details of our method in Section III. Experimental results are presented in Section IV. Finally, we summarize the discussion and conclusions of this paper in Sections V and VI.

## II Related Work

### A AD Diagnosis and Prognosis

Early diagnosis of AD is a complex process that often requires multi-modal data. Multi-modal data in AD diagnosis typically includes medical imaging (commonly sMRI and PET), genetic information, clinical data, and psychological assessments [26].

#### 1) Single-modal-based Methods

A large number of methods based on CNNs and ViTs have been proposed to achieve AD diagnosis and prognosis using only single modality data, such as MRI or PET scans. These single modality-based methods focus on identifying AD-related structural and functional changes in the brain using individual patch-level, slice-level, and voxel-level inputs [27]. However, employing unimodal neuroimaging data fails to offer a comprehensive and reliable diagnosis of AD. Previous studies have demonstrated that multi-modal neuroimaging data provides complementary information from different perspectives, allowing for improved accuracy in the early diagnosis of AD.

#### 2) Multi-modal-based Methods

Recent advancements in multi-modal methods have significantly enhanced AD diagnosis. Some studies [28], [29] utilize 3D CNNs to extract spatial features from multi-modal neuroimaging data for AD classification. However, CNNs struggle with global information modeling due to their local receptive fields. Despite these advancements, robust modality-shared representation remains challenging due to differing image scales and resolutions. To address this issue, researchers attempt to design subtle multimodal interaction techniques to enhance feature fusion for better AD diagnosis and prognosis, such as multi-modal correlation [30] and cross-attention modules [31]. Additionally, graph-based methods using multi-modal neuroimaging and non-imaging data show promise for AD prediction. However, most graph-based approaches [32], [33] require extracting features from regions-of-interest and designing feature selection algorithms, adding complexity, computational overhead, and potential information loss. Scalability issues and reliance on the limited number of labeled data further limit their efficiency and applicability. To address these limitations, recent studies [34], [35] have explored SSL techniques, such as MAE and variational autoencoders (VAEs), to learn generalizable representations from unlabeled data.

### B Vision Foundation Models in Medical Fields

In recent years, foundation models have shown promise in computer vision, particularly for natural image analysis. For instance, the Segment Anything Model (SAM) [36] and SAM2 [37] have demonstrated remarkable zero-shot image segmentation performance on unseen datasets using prompt engineering. Inspired by these advancements, researchers have turned their attention to the medical fields, such as Med-SAM [38] for medical image segmentation. However, Med-SAM’s reliance on extensive labeled data limits its clinical application. To address this, researchers are developing task-agnostic foundation models that learn generalizable modality-specific representations from large-scale unlabeled datasets using SSL strategies. These models are tailored to specific pathologies and data modalities, including retinal images [13], ultrasound [20], whole-slide images (WSIs) [17], computed tomography (CT) [21], endoscopy [39], and X-ray images [40]. These approaches have shown significant generalization ability and performance in adapting to various downstream tasks such as segmentation and classification for different organs and diseases. However, relying on single-modal imaging data can limit the ability of vision foundation models to capture comprehensive information, potentially leading to less accurate predictions [41]. Therefore, developing multi-modal vision foundation models that integrate and analyze data from various imaging modalities holds great promise for advancing generalized AI tools for the diagnosis and prognosis of AD.

### C Structured State Space Sequence Models (SSMs)

Recently, Structured State Space Sequence Models (SSMs) have shown great efficiency in modeling long-range dependencies of language sequences [42]. Subsequently, Mamba based on a selection mechanism (S6) and hardware-ware algorithm enables faster inference and linear scaling with sequence length. Vim [43] built the first pure SSM-based model with bidirectional Mamba blocks for generic tasks in computer vision and showed higher performance and significantly improved computation & memory efficiency compared to ViT-based counterparts. This success has activated further research into the application of Mamba in medical image segmentation and classification, such as U-Mamba [44], SegMamba [45], Swin-UMamba [46], and MedMamba [47]. Beyond single-modal methods, Mamba was also widely applied to multi-modal learning, including multi-modal image segmentation [48], fusion [49], and visual-language models [50]. These advancements show the potential of Mamba to be an efficient backbone for the vision foundation model in multi-modal learning for disease prediction, offering valuable insights into its capabilities and applications.

## III Method

**Fig. 1:**
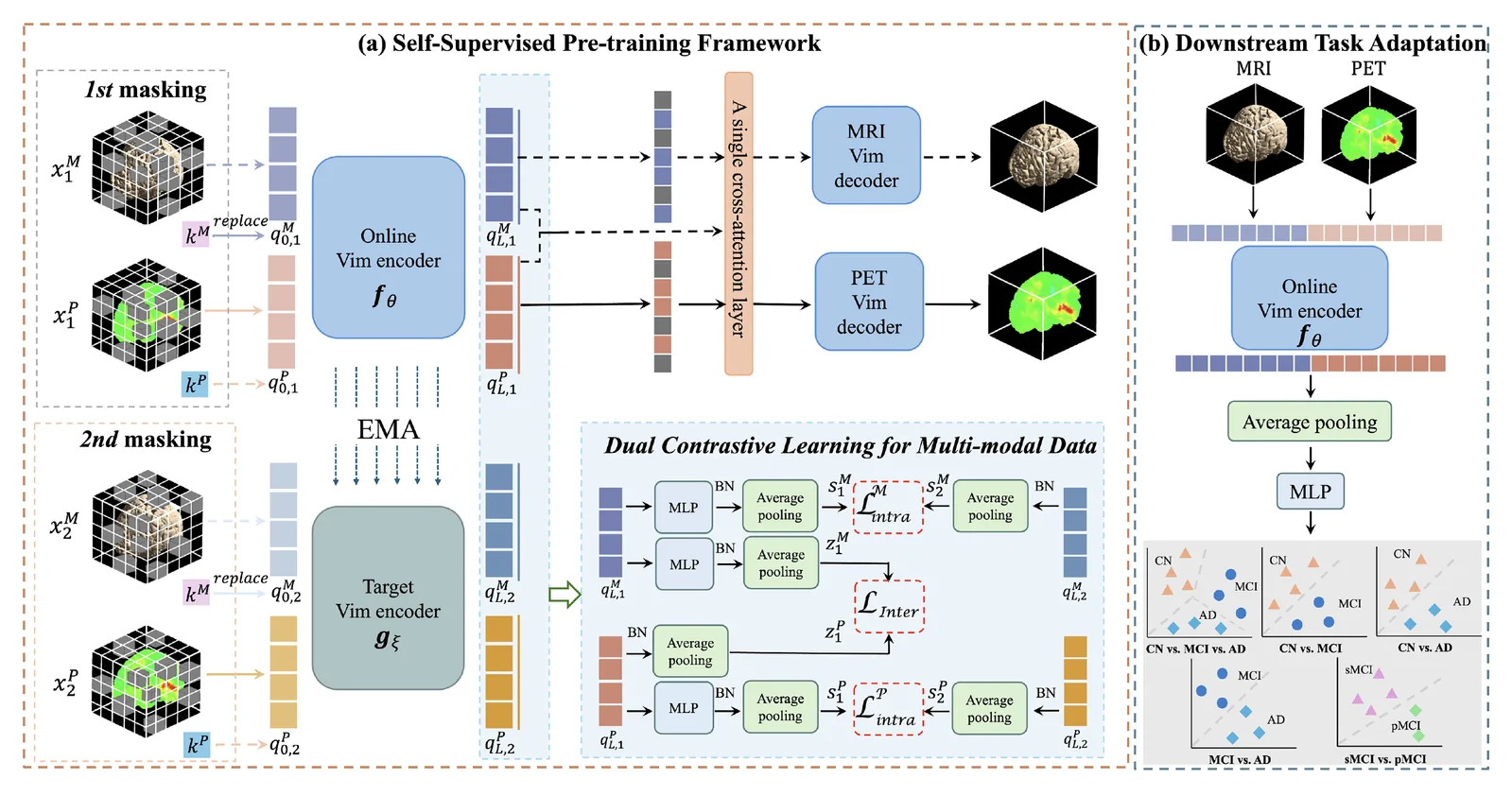
Overview of our proposed ADFound can be divided into two stages: Self-supervised pre-training and downstream task adaptation. During the self-supervised pre-training stage, we perform two rounds of random masking on 3D image patches from MRI and PET scans, generating two sequences of multi-modal patch embeddings for the unmasked patches. The learnable modality-specific embeddings ***K***^***M/P***^ are used to impute the patch embeddings of missing modality. These sequences are then input into the online Vim encoder ***f***_***θ***_ and the target Vim encoder ***g***_***ξ***_, the latter of which is updated using the exponential moving average (EMA) of the ***f***_***θ***_. The encoded token sequences from both encoders are leveraged for image reconstruction and dual contrastive learning on multi-modal data. Inter-modal contrastive learning is applied exclusively to paired multi-modal data, while the image reconstruction loss is computed using only the available modalities. The pre-trained online Vim encoder for downstream task adaptation is fine-tuned for various specific tasks.

The overall architecture of the proposed ADFound is depicted in Fig. 1. ADFound can be divided into a self-supervised pre-training framework and downstream task adaptation. ADFound employs multi-modal Vim encoders to model long-range global information of multi-modal neuroimaging data. The self-supervised pre-training framework consists of a multimodal MAE and a dual contrastive learning module for multi-modal data to pre-train ADFound on unlabeled paired and unpaired multi-neuroimaging data. For downstream task adaption, the learned online Vim encoder is fine-tuned to various AD diagnosis and prognosis tasks. For clarity, we list the main notations in Table I.

### A Image Patch Embedding

For multi-modal learning, we first transform 3D T1-MRI ***x***^*M*^ ∈ ℝ ^*C*×*D*×*W* ×*H*^ and PET images ***x***^*P*^ ∈ ℝ^*C*×*D*×*W* ×*H*^ into non-overlapping flattened MRI image patches $\{\upsilon_{j}^{M}\in {{\mathbb{R}}^{O^{3}\times C}\}}_{j=1}^{J}$ and PET image patches ${\left\{\upsilon_{j}^{P}\in {\mathbb{R}}^{O^{3}\times C}\right\}}_{j=1}^{J}$ where (*D*, *H*, *W*) represents the image resolution, *C* is the number of channels, *O* is the size of 3D image patches, and *J* is the total number of patches. we then project the image patches into a K-dimensional embedding space with linear projection matrices $W^{M}\in {\mathbb{R}}^{\left(O^{3}\cdot C\right)\times K}$ and $W^{P}\in {\mathbb{R}}^{\left(O^{3}\cdot C\right)\times K}$ and corresponding modality-specific 1-D learnable positional embeddings $E_{pos\;}^{M}\in {\mathbb{R}}^{J\times K}$ and $E_{pos\;}^{P}\in {\mathbb{R}}^{J\times K}$, as follows:

(1) $$q_{0}^{M/P}=\left[\upsilon_{0}^{M/P}W^{M/P};\ldots ;\upsilon_{J}^{M/P}W^{M/P}\right]+E_{pos\;}^{M/P},$$

where $q_{0}^{M}$ and $q_{0}^{P}$ are the embedded tokens for MRI and PET images.

### B Multi-modal Vim Encoder

Building an efficient backbone encoder is crucial to designing a robust AD foundation model. Although ViTs have been widely applied to multi-modal applications, the computational quadratic complexity of the attention mechanism and label-inefficient nature limits their capability of modeling long-range visual dependencies in medical domains [46]. Therefore, our ADFound designs a multi-modal Vim encoder based on Vim blocks [51] to model global visual context in image sequences of MRI and PET images. Vim not only shows remarkable memory efficiency and computation efficiency in visual sequence modeling but also achieves a competitive model performance compared to ViT-based counterparts, which provides valuable insights in MIM pretraining and enables multimodal tasks.

The multi-modal Vim encoder takes the multi-modal embedded token sequence as its input. Specifically, the embedded token sequences for MRI $q_{0}^{M}$ and PET $q_{0}^{P}$ are concatenated to form input token sequences ***Q***_0_ that then are fed into the multi-modal Vim encoder with *L* Vim blocks:

(2) $$Q_{0}=\left[q_{0}^{M},q_{0}^{P}\right],q_{l}=\text{Vim}\left(Q_{l-1}\right)+Q_{l-1},\;l=1\ldots L$$

(3) $$\left[q_{L}^{M},q_{L}^{P}\right]=Q_{L},$$

where $q_{L}^{M}$ and $q_{L}^{P}$ represent the encoded token sequences for MRI and PET modality.

To incorporate unpaired multi-modal data into the multi-modal Vim encoder to enhance its robustness and generalization, we addressed the challenge of unpaired data by formulating it as a missing modality problem. Specifically, the patch embeddings $q_{0}^{M/P}$ corresponding to the missing modality were replaced with modality-specific learnable embeddings ***k***^*M/P*^, which were concatenated with the patch embeddings $q_{0}^{M/P}$ of available modalities and input into the multi-modal Vim encoder.

Thanks to bidirectional SSMs in Vim blocks of the multi-modal Vim encoder, the modality-dependent global long-range context from two modalities can effectively interact with each other, resulting in cross-modal enhanced features $q_{L}^{M}$ and $q_{L}^{P}$. Meanwhile, the linear scaling property of Mamba dramatically reduces the computational costs of pre-training ADFound for 3D multi-modal representation learning.

### C Multi-modal MAE

MAE is a simple and efficient SSL framework and has achieved great success in pre-training ViT-base foundation models on large-scale unlabeled datasets. The training objective is to reconstruct masked image patches by using a small proportion of visible image patches. In this way, the model can learn complex visual representations on unlabeled data.

As shown in Fig. 1, the Multi-modal MAE adopts the multi-modal Vim encoders for encoding projected token sequences of unmasked patches to latent representations and modality-specific Vim decoders for reconstructing input images. In particular, we divide input 3D multi-modal images into non-overlapping image patches and randomly mask out the same proportion of image patches for each modality. The remaining patches from MRI and PET are projected into patch embeddings and concatenated to input into the online Vim encoder ***f***_*θ*_, generating two encoded token sequences, $q_{L,1}^{M}$ and $q_{L,1}^{P}$.

To reconstruct masked image patches, we design two modality-specific decoders based on shallow decoders with a single Vim block with the same architecture but unshared weights. To start with, the encoded token sequences $\left[q_{L,1}^{M},q_{L,1}^{P}\right]$ output from the online Vim encoder were concatenated with a set of learnable token sequences corresponding to masked patch embeddings. To integrate cross-modality information into learnable token, a single cross-attention layer views encoded single-modality tokens as queries and all tokens as keys and values following [52]. Finally, modality-specific tokens output by a single cross-attention layer were input into a lightweight decoder with a Vim block followed by an MLP layer to reconstruct the pixels. The multi-modality mamba for 3D image reconstruction was supervised by two mean square error loss ${ℒ}_{rec\;}^{M}$ and ${ℒ}_{rec\;}^{P}$ to compare the difference reconstructed pixels and the original pixels of masked image patches for T1-MRI and PET images respectively:

(4) $${ℒ}_{rec}^{M/P}=-\frac{1}{N_{M/P}}\sum {\left(y_{M/P}-{\hat{y}}_{M/P}\right)}^{2},$$

where *N*_*M/P*_ denotes the number of pixels of masked patches in MRI and PET images. *y*_*M/P*_ and *ŷ*_*M/P*_ respectively represent the pixels of reconstructed masked patches and the original masked patches.

### D Dual Contrastive Learning for Multi-modal Data

Despite MAE ensuring the network learns visual representations across modalities, it mainly focuses on modeling local spatial relations in images, neglecting relations between images in the same modality and feature misalignment between modalities in the same patients. To address this issue, we propose an intra-modal contrastive learning module to encourage our multi-modal Vim encoder to learn similar and dissimilar visual representations for the same modality. Meanwhile, to relieve the misalignment between T1-MRI and PET images, we use an inter-modal contrastive learning module to reduce the representation difference from paired T1-MRI and PET images.

#### 1) Intra-modal Contrastive Learning Module

The first step of contrastive learning is constructing positive and negative sample pairs. For MAE, it is reasonable that MAE is supposed to generate similar visual representations for two sequences of unmasked embedded token sequences from the same image after randomly masking two times, generating distinctive features for different images of the same modality. Therefore, this module introduces an additional target Vim encoder ***g***_*ξ*_ that interacts and learns mutually with the online Vim encoder to provide contrastive supervision for images in the same modality. We aim to train the online Vim encoder to capture intra-modal discriminative features effectively. The network parameter *ξ* of target Vim encoder ***g***_*ξ*_ is updated by using the EMA of parameter *θ* of online Vim encoder ***f***_*θ*_, calculated as

(5) ξ=β×ξ+(1−β)×θ,

where *β ∈* [0, 1] is a momentum coefficient.

Considering two sequences of unmasked embedded tokens $q_{0,1}^{M/P}$ and $q_{0,2}^{M/P}$ from the same image. The two sequences are individually fed into the online Vim encoder ***f***_*θ*_ and target Vim encoder ***g***_*ξ*_, generating encoded token sequences $q_{L,1}^{M/P}$ and $q_{L,2}^{M/P}$. Then we apply a batch normalization and global average pooling layer to $q_{L,1}^{M/P}$ and $q_{L,2}^{M/P}$. A projector *𝒫* with two MLP layers is only applied to the online branch, as follows:

(6) $$q_{L,1}^{M/P},q_{L,2}^{M/P}=f_{\theta }\left(q_{0,1}^{M/P}\right),g_{\xi }\left(q_{0,2}^{M/P}\right),$$

(7) $$s_{1}^{M/P},s_{2}^{M/P}=\text{𝒫}\left(\;Mean\;\left(q_{L,1}^{M/P}\right)\right),\;Mean\;\left(q_{L,2}^{M/P}\right).$$

We then calculate the similarity between $s_{1}^{M/P}$ and $s_{2}^{M/P}$, as follows:

(8) $$\rho_{M/P}=\frac{s_{1}^{M/P}\cdot s_{2}^{M/P}}{||s_{1}^{M/P}|{|}_{2}\cdot ||s_{2}^{M/P}|{|}_{2}}.$$

Therefore, InfoNCE loss (Eq.(7)) is adopted to calculate inter-modal contrastive loss, as follows:

(9) $${ℒ}_{intra\;}^{M/P}=-\log\frac{\rho_{M/P}^{+}/\tau }{\exp\left(\rho_{M/P}^{+}/\tau \right)+\sum_{i=1}^{B-1}\left(\exp\left(\rho_{M/P,i}^{-}/\tau \right)\right)},$$

where *ρ*^+^ refers to positive pairs of cosine similarity from the same image and $\rho_{i}^{-}$ indicates the cosine similarity of *i*-th negative pairs from the different images in a batch with a batch size of B. *τ* is a temperature hyper-parameter.

#### 2) Inter-modal Contrastive Learning Module

To align representations between paired T1-MRI and PET, the encoded token sequences from the online Vim encoder, denoted as $q_{L,1}^{M}$ and $q_{L,1}^{P}$, are input into batch normalization layer and a global average pooling layer, with an additional projector *𝒫* for $q_{L,1}^{M}$, generating ***z***^*M*^ and ***z***^*P*^, as follows:

(10) $$z^{M},z^{P}=\text{𝒫}\left(Mean\left(q_{L,1}^{M}\right)\right),Mean\left(q_{L,1}^{P}\right).$$

Cosine similarity between modalities is computed as follows:

(11) $$\chi =\frac{z^{M}\cdot z^{P}}{||z^{M}|{|}_{2}\cdot ||z^{P}|{|}_{2}}.$$

InfoNCE loss using in inter-modal contrastive learning can be represented as follows:

(12) $${ℒ}_{inter\;}=-\log\frac{\chi^{+}/\tau }{\exp\left(\chi^{+}/\tau \right)+\sum_{i=1}^{B-1}\left(\exp\left(\chi_{i}^{-}/\tau \right)\right)},$$

where *χ*^+^ refers to positive pairs of cosine similarity between MRI and PET from the same sample and $\chi_{i}^{-}$ indicates the cosine similarity of *i*-th negative pairs from the different samples in a batch. B and *τ* respectively signify the batch size and temperature constant.

### E Downstream Task Adaptation

After pre-training, ADFound can be easily fine-tuned into various downstream tasks for AD diagnosis and prognosis, including CN vs. MCI vs. AD, CN vs. MCI, MCI vs. AD, CN vs. AD, and sMCI vs. pMCI. The pre-trained online Vim encoder takes concatenated sequences of multi-modal image patches without random masking as its inputs. By combining a global average pooling layer and an MLP layer, ADFound can transfer learned prior knowledge from unlabeled multi-modal data into the downstream tasks. Fine-tuning ADFound is promising for reducing data dependency for downstream tasks and enhancing the model’s generalization ability across various tasks.

### F Overall Training Objective

#### 1) Pre-training

We employ a similar two-stage training strategy to CMITM [53] due to the difficulty of simultaneously optimizing reconstruction loss and inter-modal contrastive learning loss, which was observed in the previous works [53], [54]. In the first pre-training stage, the ADFound is pre-trained under the supervision of reconstruction loss for MRI ${ℒ}_{rec\;}^{M}$ and PET reconstruction ${ℒ}_{rec\;}^{P}$ and intra-modal contrastive loss for MRI $L_{intra\;}^{M}$ and PET $L_{intra\;}^{P}$, as follows:

(13) $${ℒ}_{1st}={\text{𝟙}}_{M}{ℒ}_{rec\;}^{M}+{\text{𝟙}}_{P}{ℒ}_{rec\;}^{P}+\alpha \left({\text{𝟙}}_{M}L_{intra\;}^{M}+{\text{𝟙}}_{P}L_{intra\;}^{P}\right),$$

where *α* is a hyper-parameter and 1_*M/P*_ are indicator variables that indicate whether modalities are missing, as follows:

(14) $${\text{𝟙}}_{M/P}=\{\begin{array}{cc} 1, & \text{if}\;\text{data}\;\text{is}\;\text{available,}\\ 0, & \text{if}\;\text{data}\;\text{is}\;\text{missing}.\; \end{array}$$

In the second pre-training stage, the inter-modal contrastive loss was combined into Eq. 15 with a constant weight *λ*, as follows:

(15) $${ℒ}_{2nd}={ℒ}_{1st}+\text{λ}\left({\text{𝟙}}_{M}\cdot {\text{𝟙}}_{P}\right){ℒ}_{inter\;}.$$

#### 2) Finetuning

At the stage of downstream task adaptation, the pre-trained ADFound is further fine-tuned to a wide range of AD-related downstream tasks under the supervision of cross-entropy losses for both multi-class and binary classification, as follows:

(16) $${ℒ}_{ce}=-\sum p(x)\log q(x),$$

where *p*(*x*) and *q*(*x*) respectively denotes ground truth and predicted probability.

## IV Experiments and Results

### A Dataset Description

This study was conducted on the Alzheimer’s Disease Neuroimaging Initiative (ADNI) dataset [55], including ADNI-1, ADNI-GO, ADNI-2, and ADNI-3, and OASIS-3 [56]. ADNI is a landmark research project that aims to provide a better understanding of AD through the collection and analysis of comprehensive data. We collected a total of 4362 T1-MRI scans and 2421 FDG-PET scans from the ADNI dataset for model pretraining. Specifically, 2280 MRI scans and 1740 PET scans were collected from ADNI-1 across 4.06±1.58 visits, 327 MRI scans and 276 PET scans were collected from ADNI-GO across a single visit, and 1755 MRI scans and 405 PET scans were collected from ADNI-3 across 1.71±0.91 visits. Among these, a total of 1399 MRI-PET pairs were collected from ADNI1-1, ADNI-GO, and ADNI-3 by traversing all available longitudinal time points. The detailed demographic information of data for pre-training can be found in Table II. We evaluate the performance of ADFound on ADNI-2 for a wide range of downstream tasks in AD diagnosis and prognosis, including CN vs. MCI vs. AD, CN vs. MCI, CN vs. AD, MCI vs. AD, and sMCI vs. pMCI. Following [57], we define pMCI as patients who will develop AD within 36 months, while sMCI patients will remain stable. We randomly divide the collected samples corresponding to the task into a training set (70%), a validation set (10%), and a test set (20%) in the subject level, which make sure that the samples from the same patients will be assigned to the same subset to avoid data leaky. The demographic information of collected ADNI-2 is shown in Table III. For external validation, we collected 120 samples with paired MRI-PET scans from OASIS-3 across 1.13 ±0.34 visits, as shown in Table IV.

To investigate the effectiveness of non-image data for AD diagnosis and prognosis, and to validate the flexibility of ADFound for incorporating additional modalities, we further collected several non-image data from the ADNI. These include demographic information (age, gender, education level, marital status), genetic data (APOE4 status), and neuropsychological assessments such as ADNI EF (executive function), ADNI MEM (memory performance), MMSE (Mini-Mental State Examination). These data types were selected to complement imaging biomarkers by providing insights into static risk factors, like genetic predisposition, and dynamic measures of cognitive function, enabling a more comprehensive and robust analysis of AD pathology and progression.

### B Data Pre-processing

Our data pre-processing process can be divided into several steps: 1) We randomly sample some subjects and register their PET images to the corresponding T1-MRI images using FreeSurfer [58], from which the best one is chosen as the template. 2) All T1-MRI and FDG-PET images are then registered to this template. 3) SynthStrip [59] is finally applied to perform skull stripping for all images. 4) To remove unnecessary background regions, we crop each image to the smallest 3D bounding box that contains all non-zero voxels, determined by identifying the minimum and maximum indices of non-zero regions along each axis. This step ensures that only the relevant brain region is retained, reducing the size of the data while preserving all meaningful information. 5) After cropping, we apply a two-step normalization process: First, the non-zero voxels in each image are normalized using z-score normalization, where the mean and standard deviation are calculated only from the non-zero voxels to avoid the influence of background regions. Second, for zero or near-zero voxels (background regions), their values are replaced with random samples drawn from a standard Gaussian distribution to prevent uniform zero values from biasing the downstream models. Finally, all cropped and normalized T1-MRI and FDG-PET images are resized into dimensions of 128×128×128.

### C Implementation Details

#### 1) Pre-training

The experiments were conducted on Ubuntu 22.04 using an NVIDIA GTX 4090 GPU with 24 GB VRAM. ADFound is pre-trained with 1, 600 epochs for the first pretraining stage and 500 epochs for the second pre-training stage using a batch size of 48 and an AdamW optimizer [60] with a cosine decaying learning rate and a weight decay of 0.05. The initial learning rates of the first and second pre-training stages are set to 0.005 and 0.001 respectively. The temperature parameter *τ* in Eq.9 and Eq.12 are fixed to 0.07 [61]. The hyper-parameters of weighting training losses, *α*, and *λ*, are set to 0.05 empirically. Following [61], the momentum coefficient *β* is fixed to 0.999. We used data augmentation such as random flip at the axial view and random rotation in an angle range of 0 to 10 degrees at the axial and sagittal views.

#### 2) Finetuning

The pre-trained ADFound is further finetuned to a wide range of AD-related downstream tasks, which is fine-tuned for 100 epochs using an AdamW optimizer with an initial learning rate of 0.0002 and a weight decay of 0.05. The batch size is set to 8. In addition, we follow the data augmentation method at the pre-training stage.

### D Comparison with State-of-the-art Methods

In this subsection, we first compare with the current state-of-the-art single- and multi-modal methods.

#### 1) Benchmarking Methods

We conducted extensive comparisons with nine various benchmarking methods, including generic methods [62]–[64], single-modal methods [67], [68], and multi-modal methods [31], [52], [69]. Specifically, **3D ResNet-50** [62] is a general architecture for 3D data classification. **MedicalNet** [63] is developed for 3D medical data segmentation. In this experiment, we finetune the trained MedicalNet by replacing the segmentation head in MedicalNet with an average pooling layer and a classification head for AD prediction. **ViT-3D** [64] is a 3D version of a vision transformer for classification. **Zhao et al**. [65] constructed a 3D DenseNet-based multi-classification model to achieve AD prognosis. **Zeng et al**. [66] proposed a deep belief network-based (DBN) multi-task learning for diagnosis based on ROI features extracted from modulated gray matter segmentation. **M3T** [67] is a single-modal classification network for AD diagnosis. **nnMamba** [68] integrates the strengths of CNNs and the advanced long-range modeling capabilities of State Space Sequence Models for single-modal 3D image diagnosis. **MultiMAE** [52] is a multi-modal masked autoencoder network for pre-training and followed by finetuning when applying to downstream classification tasks. **Castellano et al**. [29] proposed a multi-modal 3D CNNs designed for AD diagnosis by concatenating multi-modal featrues. **MCAD** [31] contains a multi-modal interaction module to integrate various imaging modalities and non-imaging data. We utilize only 3D MRI and PET scans as inputs for comparison. **MENet** [69] is an innovative, multi-modal lightweight network consisting of integration of spatial-wise and channel-wise enhancement blocks. In our selected comparison methods, the generic methods (ResNet-50, MeidcalNet, and ViT-3D) and single-modal methods (M3T and nnMamba) are originally designed for single-modal analysis. For a fair comparison, we concatenate the 3D MRI and PET scan along the channel dimension, forming a multi-modal input with a size of 2×128×128×128 to capture multi-modal information in AD diagnosis and prognosis.

#### 2) Model performance on downstream tasks

To validate the effectiveness of incorporating unpared data into the pertaining stage, we individually pretrain ADFound with paired-only and all multi-modal data including paired and unpaired data, and report their model performance. Our proposed ADFound is adapted to various downstream tasks in AD diagnosis and prognosis on ADNI-2, including CN vs. MCI vs. AD, CN vs. MCI, CN vs. AD, MCI vs. AD, and sMCI vs. pMCI. For each method, we repeated the experiment three times with different seeds and calculated its mean and standard errors. Following [31], Accuracy (ACC), Area Under Curve (AUC), specificity (SPE), sensitivity (SEN), and F1 score are adopted to evaluate model performance of AD diagnosis. However, for sMCI vs. pMCI classification, we replaced sensitivity (SEN) with balanced accuracy (BACC) to ensure a fair comparison due to the severe class imbalance.

**CN vs. MCI vs. AD:** The quantitative results of CN vs. MCI vs. AD classification are shown in Table V, our proposed ADFound outperforms the previous generic, single-modal, and multi-modal methods with nonnegligible improvements. Specifically, ADFound (Paired-only) achieves an average ACC and AUC of 0.617 and 0.785, which are improved by 1.8% and 1.0% compared to MCAD which has the second-best performance in multi-class classification tasks. With additional unpaired data for model pre-training, ADFound achieved a higher average ACC (0.623) and F1 score (0.641) compared to ACC (0.617) and F1 score (0.609) of the paired-only version, which highlights that leveraging unpaired data during pre-training enables ADFound to better capture diverse features from heterogeneous data distributions, leading to improved generalization and classification performance. This experiment demonstrates that the multi-class diagnostic task is highly challenging due to the overlap of symptoms, subtle differences, and the heterogeneity of AD progression. In particular, early-stage MCI patients with fewer lesions resemble CN subjects, whereas later-stage MCI patients exhibit more extensive and severe abnormalities, making them similar to early-stage AD patients. This progression complicates the multi-class diagnosis problem.

**CN vs. MCI:** Table V shows that ADFound achieves better results in most metrics for CN vs. MCI classification, yielding a higher average SPE (0.600) and F1 score (0.682) compared to the previous methods. ADFound (Paired-only) obtains the average ACC compared to the previous multi-modal methods for AD diagnosis, including the Fused Model, MCAD, and MENet, improving the ACC by 4.1%, 2.8%, and 1.1% respectively for CN vs. MCI tasks. In comparison, ADFound (All data) achieves the highest ACC of 0.652 and a SEN of 0.729, outperforming ADFound (Paired-only) in both metrics, demonstrating the benefits of incorporating unpaired data during pre-training.

**CN vs. AD:** CN vs. AD is the most common task and has been widely reported by previous studies in AD diagnosis.

It is also regarded as the simplest diagnostic task for AD diagnosis because sMRI can easily reveal significant structural changes caused by brain atrophy associated with AD progression, and FDG-PET also shows severe abnormalities in brain glucose metabolism, resulting in a conspicuous feature difference between AD patients and cognitively normal people. In Table VI, ADFound (Paired-only) dramatically improves the model performance for CN vs. AD by 2.8%, 6.5%, and 3.2% compared to MENet in terms of the average ACC, SPE, and F1 score. Additionally, ADFound (All data) further enhances performance, achieving an average ACC of 0.936 and F1 score of 0.894, outperforming both the Paired-only version and MENet.

**MCI vs. AD:** Table VI shows that ADFound achieves superior performance in classifying MCI vs. AD. ADFound (Paired-only) attains an ACC of 0.881, AUC of 0.891, SPE of 0.957, and F1 of 0.764, surpassing MCAD. In addition, ADFound (All data) further improves to an ACC of 0.899, AUC of 0.894, and SPE of 0.968, demonstrating the benefit of unpaired data. These results indicate that current models are more effective at distinguishing MCI from AD, likely due to the more pronounced differences between these stages. However, even with unpaired data, the models face greater challenges in differentiating CN from MCI, and even more so in multi-class classification among CN, MCI, and AD. This underscores the inherent complexity of early-stage cognitive diagnosis.

**sMCI vs. pMCI:** For AD prognosis, ADFound (Paired-only) yields an average ACC of 0.815 and AUC of 0.760, exceeding MENet by 2.2% and 1.3%, respectively, as shown in Table VII. In addition, ADFound (All data) achieves an improved ACC of 0.832 and AUC of 0.784, further demonstrating the benefits of incorporating unpaired data for better prognostic performance. It is worth noting that the average F1 scores of all methods are relatively low. The most plausible explanation for this issue is the pronounced class imbalance between sMCI and pMCI in the ADNI-2 dataset, with pMCI being considered as the positive sample, as illustrated in Table VII. Nevertheless, the inclusion of unpaired data allows ADFound to better handle this imbalance, resulting in more robust predictions.

**Fig. 2:**
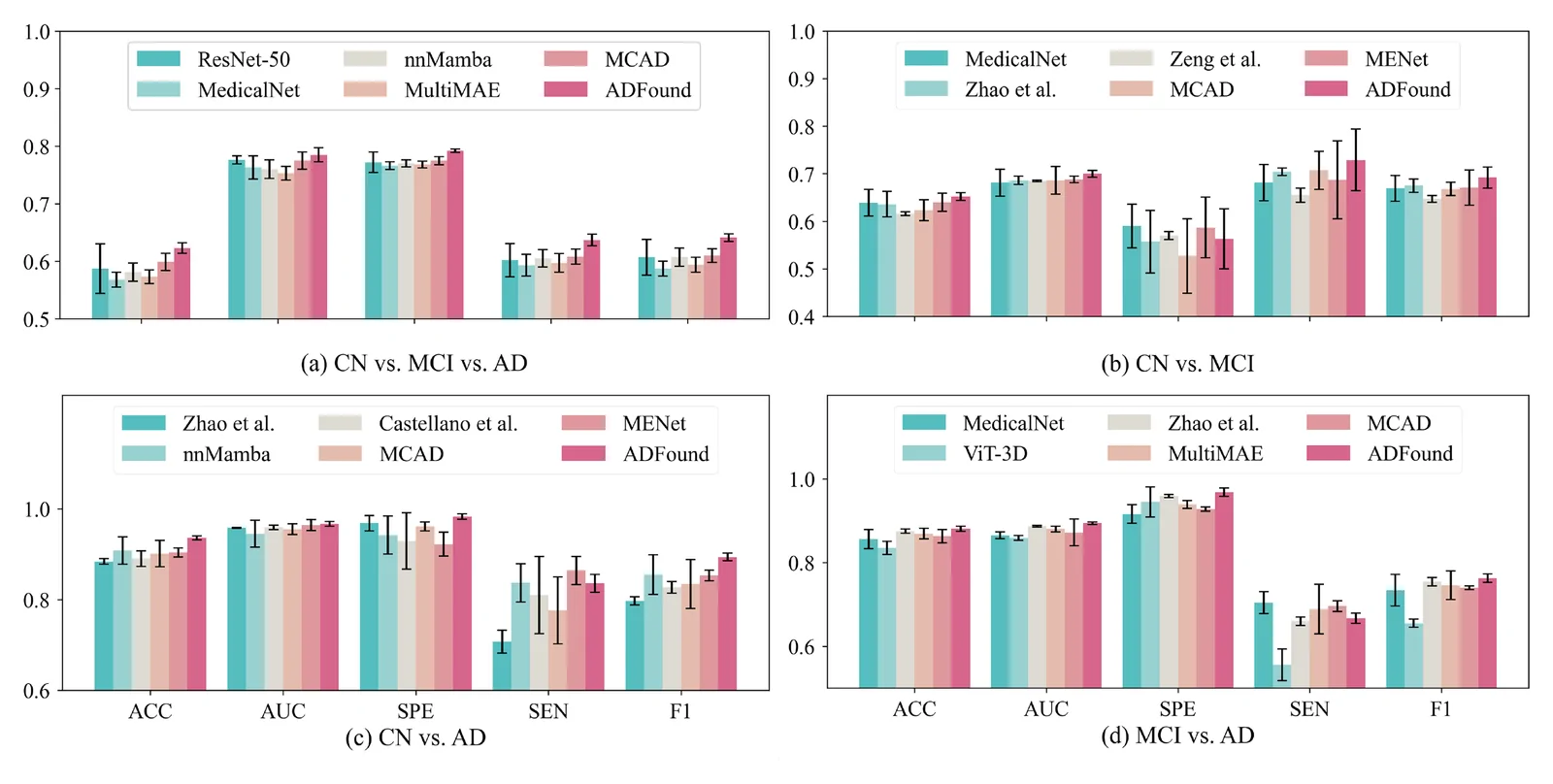
Performance comparison visualization of top five previous methods (AUC) and ADFound (All data) for CN vs. MCI vs. AD (a), CN vs. MCI (b), CN vs. AD (c), and MCI vs. AD (d).

**Fig. 3:**
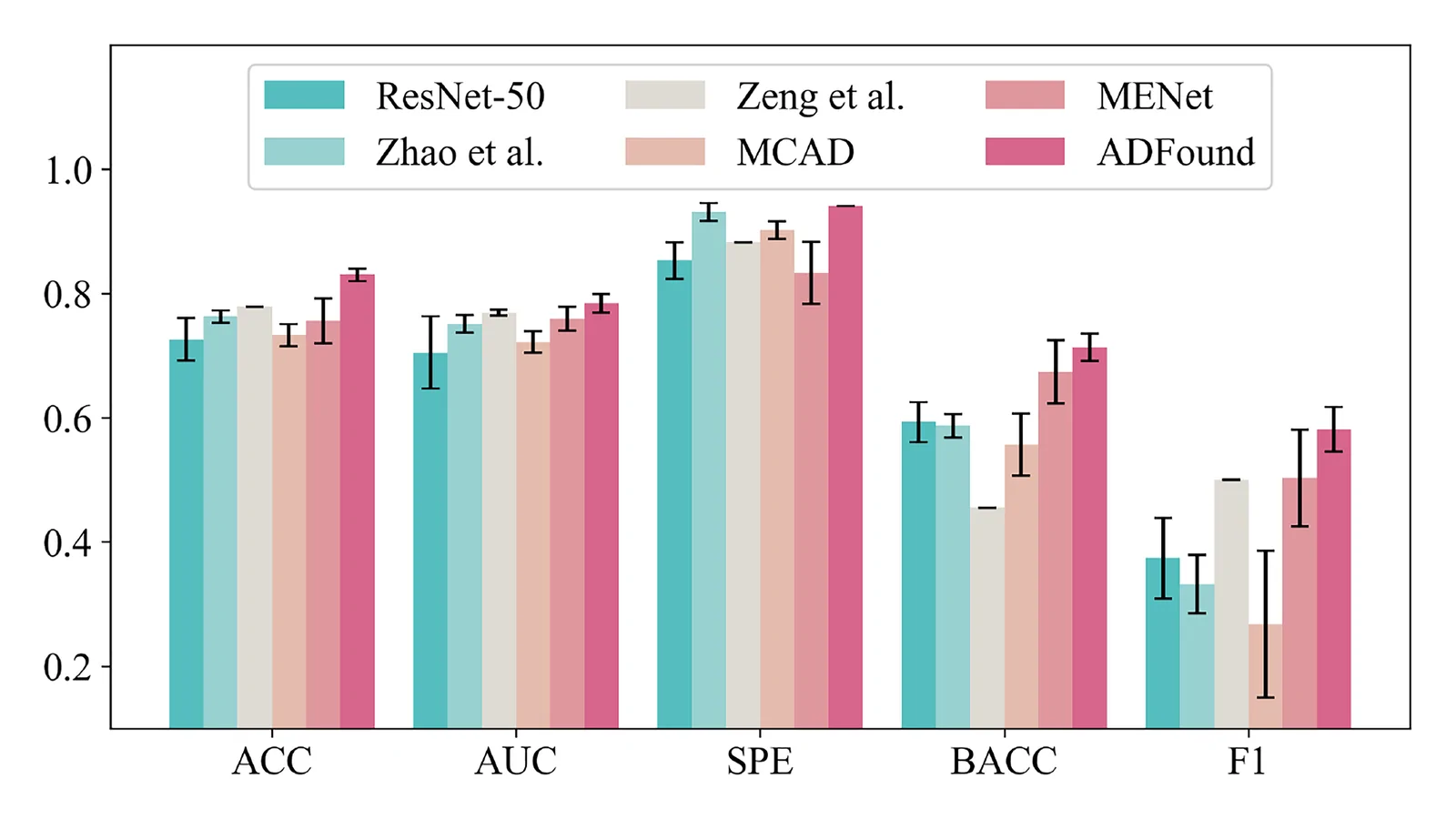
Performance comparison visualization of top five previous methods (AUC) and ADFound (All data) for sMCI vs. pMCI.

For clarity and ease of interpretation, we selected the top five previous methods for each task based on their AUC values and ADFound (All data) and visualized the results in Fig. 2 and Fig. 3. These figures demonstrate that ADFound (All data) performs better across the three primary evaluation metrics: ACC, AUC, and F1 score.

The performance metrics achieved by ADFound, while demonstrating improvements over existing methods, carry significant clinical implications, particularly in the context of AD diagnosis and prognosis. The modest accuracy and F1 scores in challenging tasks such as multi-class classification and sMCI vs. pMCI classification highlight the inherent complexity of these tasks. This complexity arises from overlapping symptoms, subtle differences, and the heterogeneity of AD progression, which can complicate accurate diagnosis and prediction. In real-world clinical settings, these limitations in diagnostic accuracy necessitate cautious interpretation of model outputs. Misclassification could lead to inappropriate treatment plans or delayed interventions, impacting patient outcomes. Therefore, it is crucial for clinicians to consider ADFound’s predictions as part of a broader diagnostic framework, integrating them with other clinical assessments and expert evaluations. Furthermore, the potential for class imbalance, particularly in the sMCI vs. pMCI task, underscores the need for continued research to enhance model robustness and reliability.

#### 3) Computation Efficiency

To demonstrate the computational efficiency of the proposed method, we provide a quantitative comparison of computational efficiency across all methods using the Multiply-Accumulate Operations (MACs) metric. MACs, measured in GMac (billion MACs), is a widely accepted standard for evaluating the computational cost of deep learning models. Lower MACs indicate reduced computational requirements, which is important for deploying models in real-time applications. Table VI shows that AD-Found (finetuning) not only outperforms other methods in terms of accuracy, but also achieves significantly lower MACs metrics (27.8 GMac) compared to other counterparts, such as MultiMAE (109.7 GMac), M3T (288.4 GMac), and MCAD (281.9 GMac). Furthermore, ADFound is also computationally efficient compared to the ROI-based DBN approach [66]. While the ROI-based method requires fewer MACs for the model itself due to its shallow architecture, it incurs significant additional computational costs during preprocessing (3.2 minutes), including modulated gray matter segmentation and ROI feature extraction. Compared to the DBN approach, ADFound only took 45ms to process each sample during inference. This efficiency makes our approach suitable for high-accuracy research applications and real-world deployment.

#### 4) External Validation

To evaluate the generalization capability of ADFound, we performed external validation on the OASIS-3 dataset for CN vs. AD classification. Despite the severe class imbalance (6 AD vs. 114 CN samples), ADFound demonstrated superior performance, achieving state-of-the-art accuracy (90.9%) and F1-score (42.8%), as shown in Table VIII. Notably, it maintained excellent sensitivity (91.3%), which is particularly valuable for clinical screening applications. However, while these results validate ADFound’s effectiveness, the relatively modest F1-score suggests that further validation on larger, more balanced datasets would be beneficial to assess its robustness in real-world clinical settings fully.

### E Ablation Study

**Fig. 4:**
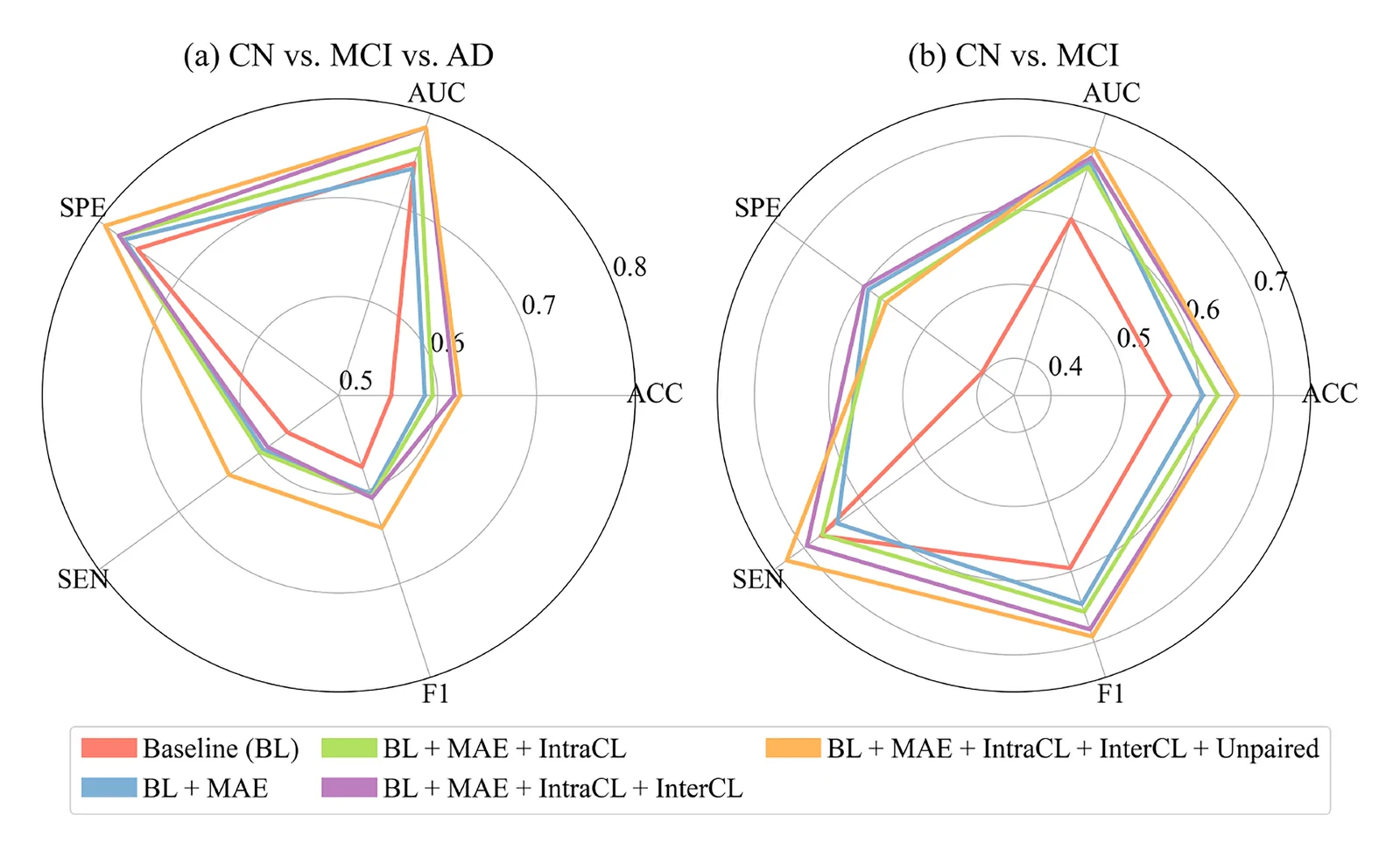
Ablation study of ADFound on CN vs. MCI vs. AD (a) and CN vs. MCI (b) classification.

We conducted an ablation study to evaluate the effectiveness of our proposed SSL strategies for multi-modal AD diagnosis. Starting with a baseline (BL) multi-modal Vim encoder trained from scratch using paired-only data, we explored the impact of incorporating Masked Autoencoder (MAE), intra-modal contrastive learning (IntraCL), inter-modal contrastive learning (InterCL), and unpaired data during pre-training. Results showed that MAE significantly improved performance, increasing ACC by 3.4% (to 0.587) and F1-score by 2.9% (to 0.605) for CN vs. MCI vs. AD, demonstrating its ability to extract meaningful features from unlabeled data. Adding IntraCL further boosted performance (ACC: 0.595), while combining MAE, IntraCL, and InterCL yielded the best results for paired data, achieving ACCs of 0.617 and 0.703 for CN vs. MCI vs. AD and CN vs. MCI, respectively. Incorporating unpaired data during pre-training resulted in the highest performance, with ACCs of 0.623 and 0.652, and F1-scores of 0.641 and 0.692 for the two tasks, highlighting the importance of leveraging unpaired data for improving generalization, feature alignment, and classification in multi-modal frameworks. For clarity and convenience, we visualize the average metrics in Fig. 4 with a radar plot.

## V Discussion

### A Comparison of ViT-based and Vim-based ADFound

**Fig. 5:**
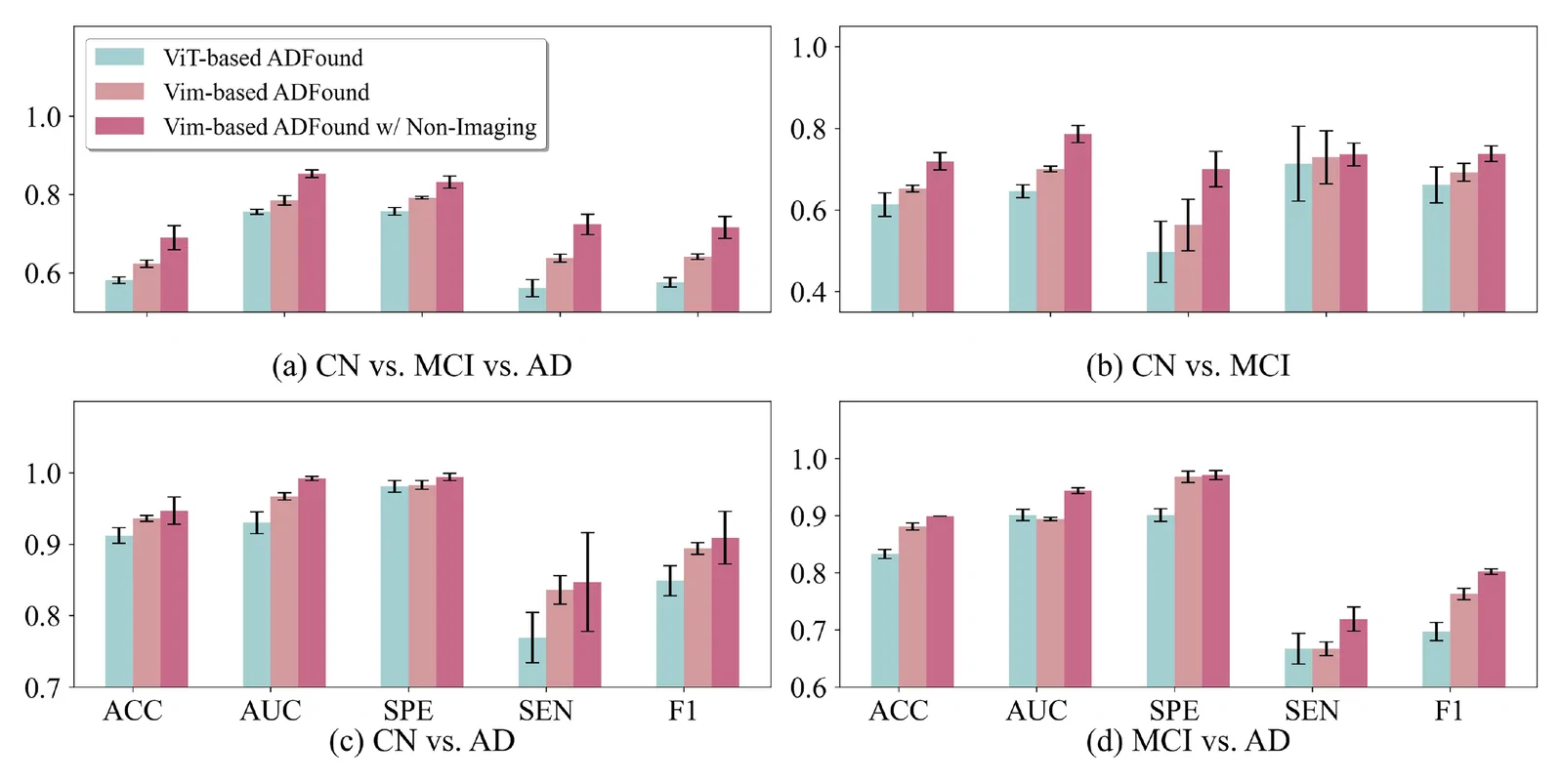
Quantitative comparison among ViT-based ADFound, Vim-based ADFound, and Vim-based ADFound with non-imaging data on multi-class (a), CN vs. MCI classification (b), CN vs. AD (c), and MCI vs. AD (d).

Benefiting from capability in long-range sequence learning and scalability to large-scale datasets and model sizes, Transformers have been successfully applied to representation learning in foundation models. Despite their revolutionary impact on handling long-range dependencies in image sequences, transformers face challenges when applied to 3D medical data. Firstly, the self-attention mechanism in Transformers, which computes pairwise interactions between all elements in the sequence and suffers from high quadratic complexity for long sequence modeling, can be computationally expensive and memory-intensive for large 3D volumes, resulting in scalability issues and limiting their applicability to high-resolution 3D medical images. Secondly, Transformers often require extensive domain-specific data to prevent overfitting and achieve competitive performance. Compared with Transformers, SSM-based methods show remarkable efficiency for long-range sequence modeling as they can scale linearly with sequence length. Therefore, we compare the performance of ViT-based and Vim-based ADFound for modeling 3D multi-modal neuroimaging data in Fig. 5 (a)-(d). Vim-based AD-Found outperforms ViT-based ADFound across all classification tasks, achieving a 4.2% higher average ACC (0.623 vs. 0.581) for CN vs. MCI vs. AD, 3.9% higher ACC (0.652 vs. 0.613) for CN vs. MCI, 2.4% higher ACC (0.936 vs. 0.912) for CN vs. AD, and 4.8% higher ACC (0.881 vs. 0.833) for MCI vs. AD, while utilizing fewer model parameters (56M vs. 93M). These results highlight the suitability of Mamba for 3D multi-modal data and its potential for efficient long-range sequence modeling.

### B Integrating Non-imaging Data into ADFound

Although ADFound focuses on 3D multi-modal neuroimaging data with paired MRI-PET scans, it can be extended to include non-imaging data, such as demographic (age, gender, education level), genetic (APOE4), and psychological assessments (MMSE, ADNI EF, ADNI MEM). We embed normalized non-imaging data into a feature vector using a fully connected layer, then concatenate it with multi-modal patch embeddings. Fig. 5 presents the classification performance of ADFound with and without non-imaging data. For CN vs. MCI vs. AD classification, incorporating non-imaging data significantly improves ACC (0.690 vs. 0.623), AUC (0.853 vs. 0.792), SPE (0.832 vs. 0.792), SEN (0.724 vs. 0.637), and F1 score (0.716 vs. 0.641). Similarly, for CN vs. MCI classification, the extended ADFound achieves higher ACC (0.719 vs. 0.652), AUC (0.786 vs. 0.700), SPE (0.700 vs. 0.563), SEN (0.736 vs. 0.729), and F1 score (0.738 vs. 0.692). For CN vs. AD classification, modest improvements are observed in ACC (0.947 vs. 0.936), AUC (0.992 vs. 0.967), SPE (0.994 vs. 0.983), SEN (0.847 vs. 0.836), and F1 score (0.909 vs. 0.894). Finally, for MCI vs. AD classification, the extended ADFound achieves higher ACC (0.899 vs. 0.881), AUC (0.944 vs. 0.894), SPE (0.971 vs. 0.968), SEN (0.719 vs. 0.667), and F1 score (0.802 vs. 0.763). These results demonstrate that integrating non-imaging data enhances AD-Found’s diagnostic performance across all classification tasks, improving accuracy, robustness, and its ability to leverage multi-modal data for AD diagnosis and prognosis.

### C Label Efficiency

**Fig. 6:**
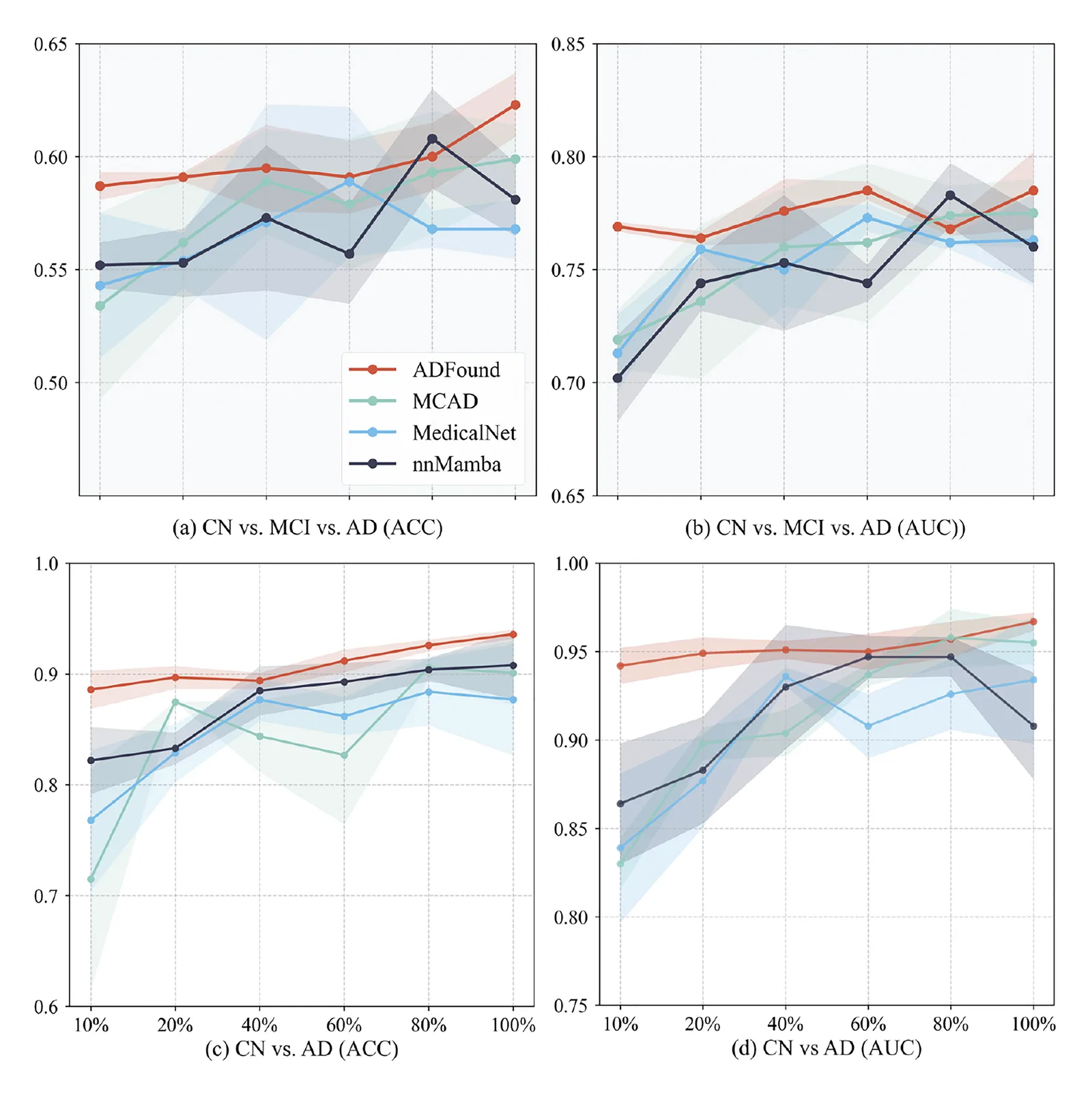
Label efficiency experiments on the CN vs. MCI vs. AD and CN vs. AD classification.

Label efficiency evaluates model performance across varying amounts of labeled data to determine the data required to reach a target accuracy. It is essential for assessing foundation models’ generalization ability under limited supervision and practical viability in data-scarce settings. Figure 6 illustrates the performance comparison between ADFound and several state-of-the-art methods, including MCAD, MedicalNet, and nnMamba, across different proportions of labeled data (10%, 20%, 40%, 60%, 80%, and 100%) for two classification tasks: CN vs. MCI vs. AD, and CN vs. AD.

Overall, ADFound consistently demonstrated superior performance, particularly when labeled data was limited. For instance, in the CN vs. MCI vs. AD classification task, ADFound exhibited a significantly smaller AUC degradation of -1.9% compared to MCAD (-5.6%), MedicalNet (-5.0%), and nnMamba (-5.8%) when trained with only 10% of the labeled data. Similarly, for the CN vs. AD classification task, ADFound achieved a much smaller AUC degradation of - 5.0%, outperforming MCAD (-18.6%), MedicalNet (-10.9%), and nnMamba (-8.6%) under the same conditions. These findings highlight ADFound’s robustness and efficiency in data-scarce settings, particularly when the availability of labeled data is limited.

### D Feature Visualization

**Fig. 7:**
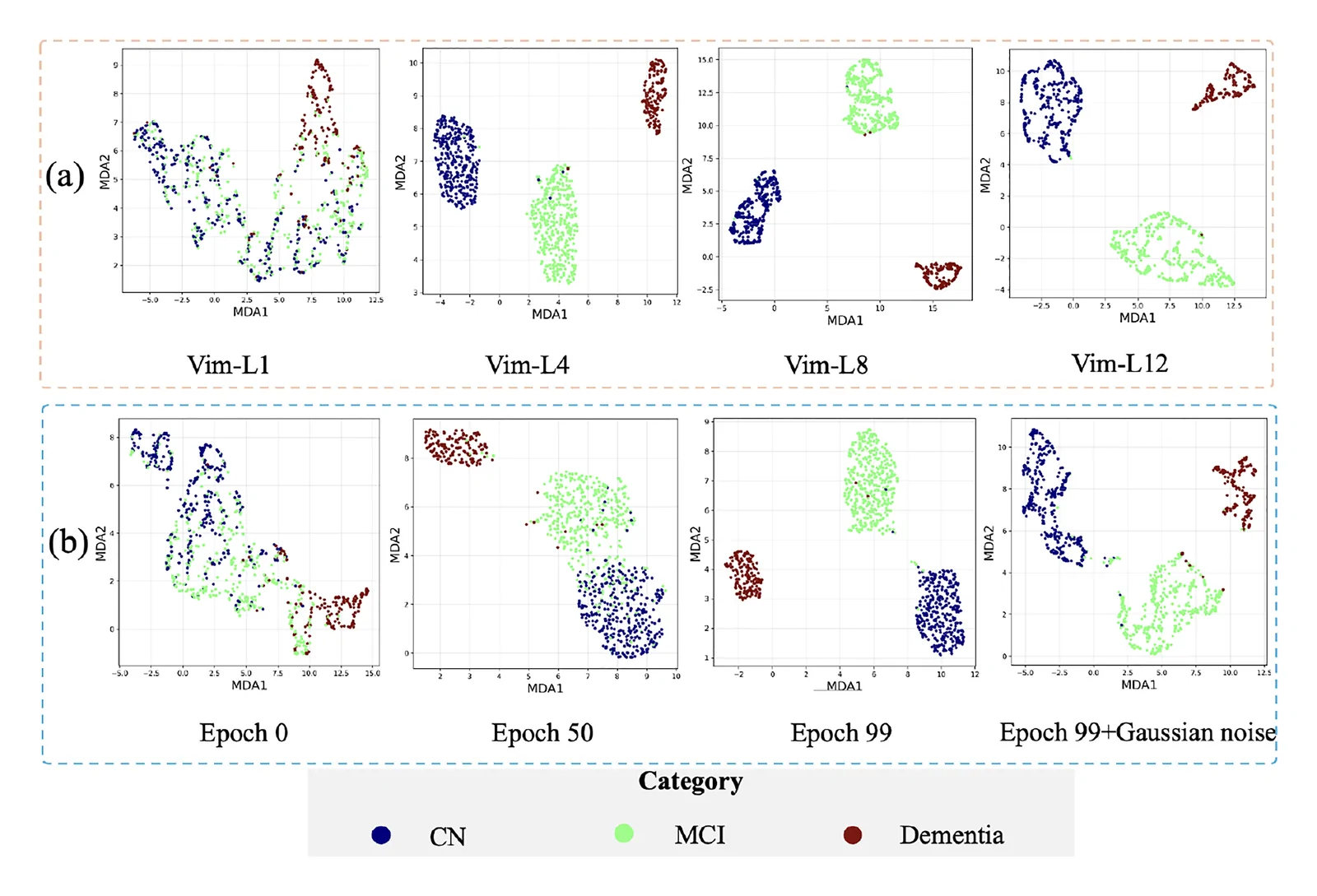
Visualization and analysis of ADFound for CN vs. MCI vs. AD classification. Vim-L1/4/8/12 represent the outputs of the 1st, 4th, 8th, and 12th Vim blocks. (a) and (b) illustrate the feature distributions of the four intermediate layers for the training and testing sets, respectively. (c) depicts the evolution of feature distributions on testing set across training epochs and the impact of adding Gaussian noise to the input data.

For feature visualization, we adopted manifold discovery and analysis (MDA) [70] to analyze the feature distribution of four intermediate layers, specifically the outputs from the 1st, 4th, 8th, and 12th Vim blocks. As shown in Fig. 7 (a), in the earlier layers (Vim-L1), the features were less structured, reflecting the raw input data with minimal transformation. As the network went deeper (Vim-L4 and Vim-L8), the features became more organized, with clusters starting to form, indicating that the network was learning meaningful abstractions. However, the training set consistently exhibited more compact and distinct clusters compared to the testing set, indicating a tendency toward overfitting. In the deeper layers (Vim-L12), the feature space was highly compact and well-separated, demonstrating the model’s ability to extract discriminative and class-specific features. Furthermore, feature distributions at the final layer evolved significantly as training epochs progressed (Fig. 7 (b)), with clusters becoming increasingly distinct and compact, reflecting the network’s ability to refine feature boundaries during optimization. When Gaussian noise was added to the input data, the feature distributions at the final layer remained robust, with well-separated and compact clusters, demonstrating the model’s ability to extract invariant and discriminative features even under noisy conditions.

### E Clinical Significance and Practical Applications

AD poses a significant clinical challenge due to its progressive nature and the complex, heterogeneous manifestations observed across patients. The multifactorial pathology of AD necessitates the integration of diverse data sources to capture subtle biological variations comprehensively. ADFound lever-ages multi-modal neuroimaging data to address the inherent complexity of AD diagnosis. By synthesizing information from various imaging modalities, ADFound aids clinicians in pinpointing subtle biomarkers that might be overlooked by traditional single-modality analyses. In addition, ADFound’s capacity to detect preclinical signs of AD enables early intervention measures. Early identification of at-risk patients allows clinicians to implement neuroprotective strategies and adjust care plans proactively. This not only optimizes patient outcomes by delaying the progression of cognitive decline but also reduces the diagnostic burden on healthcare systems by prioritizing cases that require immediate attention.

### F Limitations and Future Works

Although ADFound demonstrates promising performance in AD diagnosis and prognosis, several limitations remain. Firstly, ADFound primarily focuses on achieving AD diagnosis and prognosis using data from a single visit. However, since AD is a progressive disease, integrating longitudinal data could provide valuable insights into disease progression and improve both diagnostic accuracy and robustness. Therefore, a key direction for our future research is to extend ADFound to incorporate longitudinal data, allowing the model to capture temporal patterns and progressive changes in brain structure and function over time. Secondly, since the foundation models benefit greatly from training on large and diverse datasets, a more diverse dataset is necessary during both the pre-training and fine-tuning stages to further enhance and validate the robustness and generalization capability of ADFound. Third, more modalities should be included in the future. And the ability to handle the missing modality problem should be explored in the future.

## VI Conclusion

In this study, we introduced ADFound, the first 3D foundational model for multi-modal AD diagnosis and prognosis. ADFound employs a multi-modal Vim encoder to reduce model redundancy and computational inefficiency in ViT-based networks. We also developed a novel SSL strategy to enable ADFound to learn comprehensive multi-modal features, capturing both discriminative modality-specific and aligned modality-shared information from a large-scale unlabeled dataset with 3D paired and unpaired neuroimaging data. Our approach includes a multi-modal MAE to learn local relations among modalities through image reconstruction. Additionally, we incorporate intra-modal contrastive learning to enhance discriminative representations within the same modality and inter-modal contrastive learning to address representation mis-alignment across different modalities. We then adapted the pre-trained ADFound model for AD diagnosis and prognosis to validate its generalization ability in clinical practice. Our results demonstrate that ADFound achieves promising outcomes across various downstream tasks, underscoring its potential as a powerful tool for disease screening and health management in AD.

## Figures and Tables

**Table I:** Main Notions used in this paper

Symbol	Size	Description
***x*** ^*M/P*^	*C* × *D* × *W* × *H*	Original MRI/PET inputs
$\upsilon_{j}^{M/P}$	*O*^3^ × *C*	Non-overlapping flattened MRI/PET image patches
***W*** ^*M/P*^	(*O*^3^ · *C*) × *K*	Linear projection matrices for flattened MRI/PET im-age patches
$E_{pos\;}^{M/P}$	*J* × *K*	1-D learnable positional embedding for MRI/PET em-bedded token sequences
$q_{0}^{M/P}$	*J* × *K*	Embedded token sequences of MRI/PET
***Q***_*l*_	*J* × *K*	Token sequences output by the *l*-th Vim block
***K*** ^*M/P*^	*J* × *K*	Learnable token sequences for missing modalities
$q_{L,1}^{M/P}$	*J* × *K*	Embedded MRI/PET token sequences output by the online encoder
$q_{L,2}^{M/P}$	*J* × *K*	Embedded MRI/PET token sequences output by the Target encoder
$s_{1}^{M/P}$	1 × *K*	Normalized image features from the online encoder for intra-modal similarity calculation
$s_{2}^{M/P}$	1 × *K*	Normalized image features from the target encoder for intra-modal similarity calculation
***z*** ^*M/P*^	1 × *K*	Normalized image features for inter-modal similarity calculation
***f***_*θ*_	−	Online Vim encoder with parameter *θ*
***g***_*ξ*_	−	Target Vim encoder with parameter *ξ*
*𝒫*	−	Projector with two MLP layers
${ℒ}_{rec\;}^{M/P}$	−	Mean square error loss masked MRI/PET patches re-construction
*ρ^M/P^*	−	Intra-modal cosine similarity for MRI/PET
𝟙_*M/P*_	−	Indicator variables indicate whether MRI/PET are miss-ing
*χ*	−	Inter-modal cosine similarity
*τ*	−	Temperature hyper-parameter
*β*	−	Momentum coefficient

**Table II:** Demographic information of collected samples for model pretraining

Dataset	#MRI	#pet:	Male/Female	Age	Education	MMSE	Visit No.
ADNI-1	2280	1740	426/320	75.44±6.84	15.56±3.05	26.22±3.86	4.06±1.58
ADNI-GO	327	276	70/60	71.58±7.86	15.82±2.65	28.29±1.53	1.00±0.00
ADNI-3	1755	405	435/463	73.32±8.17	16.38±2.44	27.61±8.17	1.71±0.91
Total	4362	2421	891/799	73.75±7.55	15.99±2.76	26.73±3.71	2.78±1.81

**Table III:** Demographic information of collected samples for downstream tasks in AD diagnosis and prognosis

Category	#Samples	Male/Female	Age	Education	MMSE	Visit No.
**Diagnosis Task**						
CN	475	166/192	74.50±6.58	16.65±2.52	29.02±1.62	1.32±0.47
MCI	577	262/207	73.04±7.73	16.15±2.71	27.98±1.85	1.23±0.42
AD	239	124/100	75.25±7.89	15.89±2.67	22.00±3.95	1.14±0.34
Total	1291	528/465	73.99±7.41	16.28±2.65	27.25±3.39	1.30±0.46
**Prognosis Task**						
sMCI	169	101/68	71.53±7.47	16.22±2.78	28.23±1.67	1.00±0.00
pMCI	57	30/27	72.64±7.39	15.96±2.48	27.07±1.73	1.00±0.00
Total	226	131/95	71.81±7.47	16.16±2.71	27.94±1.76	1.00±0.00

**Table IV:** Demographic information of collected samples from OASIS-3 for external validation

Category	#Samples	Male/Female	Age	Education	MMSE	Visit No.
CN	114	42/59	67.85±7.45	16.15±2.53	29.20±1.09	1.13±0.33
AD	6	3/2	75.35±11.06	15.80±5.23	27.17±2.19	1.20±0.40
Total	120	45/61	68.22±7.84	16.13±2.72	29.10±1.25	1.13±0.34

**Table V:** Quantitative comparisons with state-of-the-art methods on ADNI-2 for CN vs. MCI vs. AD and CN vs. MCI

Method	Year	CN vs. MCI vs. AD					CN vs. MCI				
		ACC	AUC	SPE	SEN	F1	ACC	AUC	SPE	SEN	F1
ResNet-50 [62]	2018	0.587±0.043	0.776±0.007	0.772±0.018	0.602±0.029	0.607±0.031	0.583±0.024	0.623±0.053	0.470±0.173	0.681±0.105	0.635±0.021
MedicalNet [63]	2019	0.568±0.013	0.763±0.020	0.766±0.007	0.593±0.019	0.587±0.013	0.639±0.028	0.681±0.028	0.590±0.046	0.681±0.038	0.669±0.027
ViT-3D [64]	2020	0.548±0.018	0.731±0.016	0.749±0.008	0.562±0.016	0.570±0.012	0.525±0.028	0.555±0.034	0.490±0.000	0.555±0.052	0.556±0.038
Zhao et al. [65]	2021	0.518±0.006	0.734±0.007	0.739±0.004	0.548±0.008	0.543±0.007	0.636±0.027	0.686±0.009	0.557±0.066	0.704±0.008	0.675±0.014
Zeng et al. [66]	2023	0.545±0.014	0.737±0.008	0.748±0.010	0.561±0.018	0.557±0.014	0.616±0.004	0.685±0.002	0.570±0.008	0.655±0.015	0.647±0.007
M3T [67]	2022	0.474±0.051	0.655±0.090	0.704±0.034	0.444±0.089	0.434±0.099	0.551±0.016	0.560±0.025	0.450±0.092	0.638±0.049	0.604±0.010
nnMamba [68]	2024	0.581±0.016	0.760±0.016	0.770±0.006	0.605±0.015	0.607±0.016	0.613±0.010	0.661±0.005	0.565±0.064	0.655±0.037	0.645±0.007
MultiMAE [52]	2022	0.573±0.012	0.753±0.012	0.768±0.006	0.597±0.016	0.594±0.013	0.576±0.039	0.616±0.018	0.570±0.070	0.580±0.033	0.595±0.032
Castellano et al. [29]	2024	0.530±0.027	0.742±0.015	0.749±0.011	0.575±0.028	0.548±0.017	0.548±0.017	0.667±0.009	0.517±0.074	0.690±0.030	0.655±0.004
MCAD [31]	2023	0.599±0.015	0.775±0.015	0.775±0.007	0.608±0.013	0.610±0.012	0.623±0.022	0.686±0.029	0.527±0.078	0.707±0.040	0.668±0.014
MENet [69]	2023	0.552±0.023	0.738±0.031	0.753±0.013	0.571±0.027	0.573±0.022	0.640±0.019	0.688±0.007	0.587±0.064	0.687±0.082	0.671±0.037
**ADFound** (Paired-only)	ours	0.617±0.014	0.785±0.017	0.775±0.007	0.589±0.012	0.609±0.009	0.651±0.025	0.687±0.033	**0.600±0.026**	0.695±0.026	0.682±0.024
**ADFound** (All data)	ours	**0.623±0.009**	**0.785±0.012**	**0.792±0.003**	**0.637±0.010**	**0.641±0.007**	**0.652±0.008**	**0.700±0.007**	0.563±0.063	**0.729±0.065**	**0.692±0.022**

**Table VI:** Quantitative comparisons with state-of-the-art methods on ADNI-2 for CN vs. AD and MCI vs. AD.

Method	MACs(G)	CN vs. AD					MCI vs. AD				
		ACC	AUC	SPE	SEN	F1	ACC	AUC	SPE	SEN	F1
ResNet-50	93.3	0.904±0.020	0.940±0.026	0.961±0.010	0.782±0.051	0.839±0.036	0.823±0.034	0.843±0.020	0.875±0.041	0.689±0.022	0.688±0.049
MedicalNet	93.3	0.877±0.050	0.934 ±0.036	0.932±0.042	0.762±0.072	0.800±0.079	0.856±0.023	0.865±0.008	0.916±0.022	0.704±0.026	0.734±0.038
V1T-3D	51.6	0.842±0.043	0.862 ±0.038	0.968±0.011	0.578±0.116	0.698±0.102	0.835±0.016	0.859±0.006	0.945±0.036	0.556±0.038	0.655±0.010
Zhao et al.	88.0	0.884±0.006	0.958±0.001	0.968±0.017	0.707±0.025	0.797±0.009	0.875±0.005	0.887±0.002	0.959±0.004	0.660±0.010	0.754±0.010
Zeng et al.	< 0.1	0.888±0.005	0.941±0.001	0.922±0.000	0.816±0.017	0.825±0.010	0.804±0.006	0.852±0.001	0.865±0.008	0.652±0.000	0.657±0.007
M3T	288.4	0.800±0.083	0.831±0.067	0.922±0.054	0.544±0.145	0.636±0.159	0.756±0.023	0.780±0.056	0.957±0.038	0.244±0.168	0.336±0.194
nnMamba	42.9	0.908±0.030	0.945±0.030	0.942±0.042	0.837±0.042	0.855±0.044	0.842±0.016	0.858±0.006	0.922±0.036	0.637±0.038	0.693±0.010
MultiMAE	109.7	0.853±0.015	0.928±0.007	0.945±0.011	0.660±0.024	0.743±0.027	0.869±0.013	0.880±0.007	0.939±0.009	0.689±0.059	0.746±0.034
Castellano et al.	71.1	0.890±0.017	0.959±0.005	0.929±0.062	0.810±0.085	0.827±0.013	0.810±0.013	0.844±0.009	0.864±0.095	0.674±0.078	0.670±0.030
MCAD	281.9	0.901±0.029	0.955±0.012	0.961±0.010	0.776±0.074	0.834±0.054	0.863±0.016	0.872±0.032	0.928±0.005	**0.696±0.013**	0.740±0.004
MENet	141.1	0.904±0.010	0.964±0.012	0.922±0.026	**0.864±0.031**	0.853±0.012	0.871±0.047	0.844±0.009	0.864±0.028	0.674±0.078	0.670±0.030
**ADFound** (Paired-only)	27.8	0.932±0.014	0.954±0.011	**0.987±0.015**	0.816±0.035	0.885±0.024	0.881±0.011	0.891±0.007	0.957±0.009	0.689±0.059	**0.764±0.032**
**ADFound** (All data)	27.8	**0.936±0.004**	**0.967±0.005**	0.983±0.006	0.836±0.020	**0.894±0.008**	**0.881±0.006**	**0.894±0.003**	**0.968±0.010**	0.667±0.012	0.763±0.010

**Table VII:** Quantitative comparisons with state-of-the-art methods on ADNI-2 for sMCI vs. pMCI classification.

Method	sMCI vs. pMCI				
	ACC	AUC	SPE	BACC	F1
ResNet-50	0.726±0.034	0.705±0.058	0.853±0.029	0.593±0.032	0.374±0.065
MedicalNet	0.763±0.026	0.669±0.033	0.902±0.017	0.618±0.028	0.407±0.064
ViT-3D	0.741±0.026	0.537±0.060	0.873±0.045	0.603±0.014	0.385±0.034
Zhao et al.	0.763±0.010	0.751±0.014	0.931±0.014	0.587±0.019	0.332±0.047
Zeng et al.	0.778±0.000	0.769±0.005	0.882±0.000	0.455±0.000	0.500±0.000
M3T	0.733±0.031	0.628±0.155	0.926±0.062	0.531±0.001	0.195±0.058
nnMamba	0.733±0.038	0.683±0.065	0.843±0.068	0.619±0.129	0.405±0.147
MultiMAE	0.807±0.013	0.701±0.011	0.931±0.017	0.678±0.019	0.517±0.043
Castellano et al.	0.667±0.044	0.676±0.065	0.716±0.065	0.615±0.051	0.430±0.078
MCAD	0.733±0.018	0.722±0.017	0.902±0.014	0.557±0.050	0.268±0.118
MENet	0.756±0.036	0.759±0.019	0.833±0.050	0.674±0.051	0.503±0.078
**ADFound** (Paired-only)	0.815±0.046	0.760±0.011	0.912±0.029	**0.714±0.067**	0.575±0.109
**ADFound** (All data)	**0.830±0.010**	**0.784±0.015**	**0.941±0.000**	0.713±0.022	**0.581±0.036**

**Table VIII:** External validation on OASIS-3 dataset.

Method	CN vs. AD				
	ACC	AUC	SPE	SEN	F1
Zhao et al.	0.878±0.016	0.922±0.011	0.882±0.017	0.800±0.000	0.356±0.033
nnMamba	0.807±0.030	**0.936±0.028**	0.804±0.028	0.866±0.094	0.275±0.051
Castellano et al.	0.797±0.020	0.911±0.016	0.797±0.021	0.810±0.021	0.247±0.019
MENet	0.892±0.021	0.928±0.064	**0.894±0.027**	0.866±0.231	0.398±0.072
MCAD	0.818±0.008	0.904±0.009	0.818±0.008	0.832±0.012	0.267±0.008
**ADFound** (All data)	**0.909±0.024**	0.921±0.021	0.800±0.000	**0.913±0.025**	**0.428±0.068**

## References

[1] Scheltens P, De Strooper B, Kivipelto M, Holstege H, Chételat G, Teunissen CE, Cummings J, van der Flier WM (2021). Alzheimer’s disease. The Lancet.

[2] Joe E, Ringman JM (2019). Cognitive symptoms of alzheimer’s disease: clinical management and prevention. bmj.

[3] Canonici AP, Andrade LPD, Gobbi S, Santos-Galduroz RF, Gobbi LTB, Stella F (2012). Functional dependence and caregiver burden in alzheimer’s disease: a controlled trial on the benefits of motor intervention. Psychogeriatrics.

[4] Zvěřová M (2018). Alzheimer’s disease and blood-based biomarkers–potential contexts of use. Neuropsychiatr Dis Treat.

[5] Cui R, Liu M (2018). Hippocampus analysis by combination of 3-D DenseNet and shapes for Alzheimer’s disease diagnosis. IEEE Journal of Biomedical and Health Informatics.

[6] Lian C, Liu M, Zhang J, Shen D (2018). Hierarchical fully convolutional network for joint atrophy localization and alzheimer’s disease diagnosis using structural mri. IEEE transactions on pattern analysis and machine intelligence.

[7] Zhang Y, Teng Q, Liu Y, Liu Y, He X (2022). Diagnosis of alzheimer’s disease based on regional attention with smri gray matter slices. Journal of neuroscience methods.

[8] Hoang GM, Kim U-H, Kim JG (2023). Vision transformers for the prediction of mild cognitive impairment to alzheimer’s disease progression using mid-sagittal smri. Frontiers in Aging Neuroscience.

[9] Huang F, Qiu A, A. D. N. Initiative (2024). Ensemble vision transformer for dementia diagnosis. IEEE Journal of Biomedical and Health Informatics.

[10] Yu Q (2024). A transformer-based unified multimodal framework for alzheimer’s disease assessment. Computers in Biology and Medicine.

[11] Sibilano E (2024). Understanding the role of self-attention in a transformer model for the discrimination of scd from mci using resting-state eeg. IEEE Journal of Biomedical and Health Informatics.

[12] Cheung EY, Wu RW, Chu ES, Mak HK (2024). Integrating demographics and imaging features for various stages of dementia classification: Feed forward neural network multi-class approach. Biomedicines.

[13] Zhou Y (2023). A foundation model for generalizable disease detection from retinal images. Nature.

[14] Moor M, Banerjee O, Abad ZSH, Krumholz HM, Leskovec J, Topol EJ, Rajpurkar P (2023). Foundation models for generalist medical artificial intelligence. Nature.

[15] Lu MY (2024). A visual-language foundation model for computational pathology. Nature Medicine.

[16] Huang Z, Bianchi F, Yuksekgonul M, Montine TJ, Zou J (2023). A visual–language foundation model for pathology image analysis using medical twitter. Nature medicine.

[17] Chen RJ (2024). Towards a general-purpose foundation model for computational pathology. Nature Medicine.

[18] Christensen M (2024). Vision–language foundation model for echocardiogram interpretation. Nature Medicine.

[19] Kim C, Gadgil SU, DeGrave AJ, Omiye JA, Cai ZR, Daneshjou R, Lee S-I (2024). Transparent medical image ai via an image–text foundation model grounded in medical literature. Nature Medicine.

[20] Jiao J (2024). Usfm: A universal ultrasound foundation model generalized to tasks and organs towards label efficient image analysis. Medical Image Analysis.

[21] Wang G, Wu J, Luo X, Liu X, Li K, Zhang S (2023). Mis-fm: 3d medical image segmentation using foundation models pretrained on a large-scale unannotated dataset. arXiv preprint.

[22] Zhang X, Wu C, Zhang Y, Xie W, Wang Y (2023). Knowledge-enhanced visual-language pre-training on chest radiology images. Nature Communications.

[23] Xiang J (2025). A vision–language foundation model for precision oncology. Nature.

[24] Zhao T (2024). A foundation model for joint segmentation, detection and recognition of biomedical objects across nine modalities. Nature methods.

[25] Gu A, Dao T (2023). Mamba: Linear-Time Sequence Modeling with Selective State Spaces. arXiv preprint.

[26] Tang Y, Xiong X, Tong G, Yang Y, Zhang H (2024). Multimodal diagnosis model of alzheimer’s disease based on improved transformer. BioMedical Engineering OnLine.

[27] Arya AD, Verma SS, Chakarabarti P, Chakrabarti T, Elngar AA, Kamali A-M, Nami M (2023). A systematic review on machine learning and deep learning techniques in the effective diagnosis of alzheimer’s disease. Brain Informatics.

[28] Zhang F, Li Z, Zhang B, Du H, Wang B, Zhang X (2019). Multi-modal deep learning model for auxiliary diagnosis of alzheimer’s disease. Neurocomputing.

[29] Castellano G, Esposito A, Lella E, Montanaro G, Vessio G (2024). Automated detection of alzheimer’s disease: a multi-modal approach with 3d mri and amyloid pet. Scientific Reports.

[30] Zhang Y, Sun K, Liu Y, Shen D (2023). Transformer-based multimodal fusion for early diagnosis of alzheimer’s disease using structural mri and pet.

[31] Zhang J (2023). Multi-modal cross-attention network for alzheimer’s disease diagnosis with multi-modality data. Computers in Biology and Medicine.

[32] Lei B (2024). Alzheimer’s disease diagnosis from multi-modal data via feature inductive learning and dual multilevel graph neural network. Medical Image Analysis.

[33] Zhou H, He L, Chen BY, Shen L, Zhang Y (2024). Multi-modal diagnosis of alzheimer’s disease using interpretable graph convolutional networks. IEEE Transactions on Medical Imaging.

[34] Kunanbayev K, Shen V, Kim D-S (2024). Training vit with limited data for alzheimer’s disease classification: An empirical study.

[35] Martinez-Murcia FJ, Arco JE, Jimenez-Mesa C, Segovia F, Illan IA, Ramirez J, Gorriz JM (2024). Bridging imaging and clinical scores in parkinson’s progression via multimodal self-supervised deep learning. International Journal of Neural Systems.

[36] Kirillov A (2023). Segment anything.

[37] Ravi N, Gabeur V, Hu Y-T, Hu R, Ryali C, Ma T, Khedr H, Rädle R, Rolland C, Gustafson L (2024). Sam 2: Segment anything in images and videos. arXiv preprint.

[38] Ma J (2024). Segment anything in medical images. Nature Communications.

[39] Wang Z, Liu C, Zhang S, Dou Q (2023). Foundation model for endoscopy video analysis via large-scale self-supervised pre-train.

[40] Tiu E, Talius E, Patel P, Langlotz CP, Ng AY, Rajpurkar P (2022). Expert-level detection of pathologies from unannotated chest x-ray images via self-supervised learning. Nature Biomedical Engineering.

[41] Zhang S (2023). On the challenges and perspectives of foundation models for med. image anal. Med Image Anal.

[42] Gu A, Goel K, Ré C (2022). Efficiently modeling long sequences with structured state spaces.

[43] Zhu L, Liao B, Zhang Q, Wang X, Liu W, Wang X (2024). Vision mamba: Efficient visual representation learning with bidirectional state space model.

[44] Ma J, Li F, Wang B (2024). U-mamba: Enhancing long-range dependency for biomedical image segmentation. arXiv preprint.

[45] Xing Z, Ye T, Yang Y, Liu G, Zhu L (2024). Segmamba: Long-range sequential modeling mamba for 3d medical image segmentation.

[46] Liu J (2024). Swin-umamba: Mamba-based unet with imagenet-based pretraining.

[47] Yue Y, Li Z (2024). Medmamba: Vision mamba for medical image classification. arXiv preprint.

[48] Wan Z, Zhang P, Wang Y, Yong S, Stepputtis S, Sycara K, Xie Y (2025). Sigma: Siamese mamba network for multi-modal semantic segmentation.

[49] Xie X, Cui Y, Tan T, Zheng X, Yu Z (2024). Fusionmamba: Dynamic feature enhancement for multimodal image fusion with mamba. Visual Intelligence.

[50] Qiao Y, Yu Z, Guo L, Chen S, Zhao Z, Sun M, Wu Q, Liu J (2024). Vl-mamba: Exploring state space models for multimodal learning. CoRR.

[51] Zhu L, Liao B, Zhang Q, Wang X, Liu W, Wang X (2024). Vision mamba: Efficient visual representation learning with bidirectional state space model. arXiv preprint.

[52] Bachmann R, Mizrahi D, Atanov A, Zamir A (2022). Multimae: Multi-modal multi-task masked autoencoders.

[53] Chen C, Zhong A, Wu D, Luo J, Li Q (2023). Contrastive masked image-text modeling for medical visual representation learning.

[54] Jiang Z, Chen Y, Liu M, Chen D, Dai X, Yuan L, Liu Z, Wang Z (2023). Layer grafted pre-training: Bridging contrastive learning and masked image modeling for label-efficient representations.

[55] Weiner MW (2013). The alzheimer’s disease neuroimaging initiative: a review of papers published since its inception. Alzheimer’s & Dementia.

[56] LaMontagne PJ, Keefe S, Lauren W, Xiong C, Grant EA, Moulder KL, Morris JC, Benzinger TL, Marcus DS (2018). P3-083: Oasis-3: Longitudinal neuroimaging, clinical, and cognitive dataset for normal aging and alzheimer’s disease. Alzheimer’s & Dementia.

[57] Tu Y, Lin S, Qiao J, Zhuang Y, Wang Z, Wang D (2024). Multimodal fusion diagnosis of alzheimer’s disease based on fdg-pet generation. Biomedical Signal Processing and Control.

[58] Fischl B (2012). Freesurfer. Neuroimage.

[59] Hoopes A (2022). SynthStrip: skull-stripping for any brain image. NeuroImage.

[60] Loshchilov I, Hutter F (2019). Decoupled weight decay regularization.

[61] He K (2020). Momentum contrast for unsupervised visual representation learning.

[62] Hara K, Kataoka H, Satoh Y (2018). Can spatiotemporal 3d cnns retrace the history of 2d cnns and imagenet?.

[63] Chen S, Ma K, Zheng Y (2019). Med3d: Transfer learning for 3d med. image anal. arXiv preprint.

[64] Dosovitskiy A (2021). An Image is Worth 16x16 Words: Transformers for Image Recognition at Scale.

[65] Zhao Y, Ma B, Jiang P, Zeng D, Wang X, Li S (2020). Prediction of alzheimer’s disease progression with multi-information generative adversarial network. IEEE Journal of Biomedical and Health Informatics.

[66] Zeng N, Li H, Peng Y (2023). A new deep belief network-based multi-task learning for diagnosis of alzheimer’s disease. Neural Computing and Applications.

[67] Jang J, Hwang D (2022). M3T: three-dimensional Medical image classifier using Multi-plane and Multi-slice Transformer. CVPR.

[68] Gong H, Kang L, Wang Y, Wang Y, Wan X, Wu X, Li H (2025). nn-mamba: 3d biomedical image segmentation, classification and landmark detection with state space model.

[69] Leng Y (2023). Multimodal cross enhanced fusion network for diagnosis of alzheimer’s disease and subjective memory complaints. Computers in Biology and Medicine.

[70] Islam MT, Zhou Z, Ren H, Khuzani MB, Kapp D, Zou J, Tian L, Liao JC, Xing L (2023). Revealing hidden patterns in deep neural network feature space continuum via manifold learning. Nature Communications.

